# Expression of PPARα, β, and γ in the Hartley guinea pig model of primary osteoarthritis

**DOI:** 10.1186/1479-5876-10-S3-P38

**Published:** 2012-11-28

**Authors:** Fatima Ezzahra El Mansouri, Salwa Sarah Nebbaki, Nadia Zayed, Mohamed Benderdour, Johanne Martel-Pelletier, Jean-Pierre Pelletier, Hassan Fahmi

**Affiliations:** 1 Osteoarthritis Research Unit, University of Montreal Hospital Research Center (CRCHUM), Notre-Dame Hospital, Montreal, Quebec, Canada; 2 Centre de recherche-Hôpital Sacré-Coeur de Montréal, Montréal, Quebec, Canada

## Background

Peroxisome proliferator-activated receptors (PPARs) are members of the nuclear receptor superfamily. Three isoforms have been identified: PPARα, PPARβ-δ and PPARγ. Several in vitro and in vivo studies suggest that PPARγ may have protective roles in osteoarthritis (OA). So far, little is known about the pattern of PPAR expression during the progression of OA and cartilage degradation.

## Aim

To investigate the expression of PPARα, β, and γ in cartilage over the course of OA in the spontaneous Hartley guinea pig model.

## Methods

Hartley guinea pigs were sacrificed at 2 (control group), 4, 8, and 12 (n = 6 per group) month-old of age. Cartilage was obtained from the central portion of the medial tibial plateau. Cartilage degradation was evaluated histologically using the Osteoarthritis Research Society International (OARSI) guidelines. The expression of PPARα, β and γ was analyzed by immunohistochemistry. The non-parametric Spearman test was used for the correlation analysis between the protein expression levels and histological scores.

## Results

PPARα, β and γ, were detected in medial tibial plateaus from control animals. There was no significant change in the levels of PPARα and PPARβ over the course of OA. In contrast, PPARγ expression decreased during the progression of OA. Correlation analysis revealed a negative correlation between PPARγ levels and histological score of OA.

## Conclusion

Expression of PPARγ in cartilage decreased during the course of OA. These data suggest that loss of PPARγ expression in cartilage may contribute to the pathogenesis of OA.

